# Delivering Essential Surgical Care for Lower-limb Musculoskeletal disorders in the Low-Resource Setting

**DOI:** 10.1007/s00268-021-06211-3

**Published:** 2021-06-29

**Authors:** Deeptiman James, Faye M. Evans, Ekta Rai, Nobhojit Roy

**Affiliations:** 1 Pediatric Orthopedic Unit, Department of Orthopedic, Christian Medical College Vellore, 1106, Paul Brand Building, Ida Scudder Road, Vellore, 632004 India; 2 Department of Anesthesiology, Critical Care and Pain Medicine, Boston Children’s Hospital, Harvard Medical School, Boston, US; 3 Department of Anesthesia, Head of Pediatric and ObGyn Anesthesia unit, Christian Medical College, Vellore, Tamil Nadu India; 4 WHO Collaborating Center for Research on Surgical Care Delivery in LMICs, BARC Hospital, HBNI University, Mumbai, India; 5 Department of Global Public Health, Karolinska Institutet, Stockholm, Sweden; 6 Christian Hospital Mungeli, Mungeli, Chhattisgarh 495334 India

## Abstract

**Background:**

Mismatched surgeon-anesthesiologist ratios often exist in low-resource settings making safe emergency essential surgical care challenging. This study is an audit of emergency essential procedures performed for lower-limb (LL) musculoskeletal disorders (MSD) when an anesthesiologist was unavailable. It aims to identify strategies for safe anesthesia.

**Methods:**

A 5-year retrospective audit of emergency essential LL orthopedic procedures performed at remote mission hospital in Central India was performed. Out of necessity, a regional anesthesia (RA) protocol was developed in collaboration with anesthesiologists familiar with the setting. The incidence of intraoperative surgical and perioperative anesthesia complications when RA was administered by a surgeon was evaluated.

**Results:**

During this period, 766 emergency essential LL MSDs procedures were performed. An anesthesiologist was available for only 6/766. RA was administered by a surgeon for 283/766. This included spinal anesthesia (SA) for 267/283 patients, peripheral nerve blocks for 16/283. Local infiltration and/or sedation was administered to 477/766. There were 17 intraoperative surgical complications. Anesthesia-related complications included 37/267 patients who required multiple attempts to localize subarachnoid space and SA failure in 9/267 patients all of whom had successful re-administration. Additional sedation and infiltration of local anesthetic was required in 5/267 patients.

**Conclusion:**

Remote pre-anesthesia consultation for high-risk patients, local surgeon-anesthesiologist networking, protocol-guided management, and dedicated short duration of training in anesthesia may be considered as an alternative for delivering RA for emergency essential surgery for LL MSDs due to unavailability of anesthesiologists.

## Introduction

Essential surgical care for musculoskeletal disorders listed in the Disease Control Priorities, 3^rd^edition (DCP3) provides broad guidelines to define essential orthopedic care at first-level hospitals in low- and middle-income countries (LMICs) [1]. Realistically adherence to these guidelines while delivering essential surgical care is often challenging. One of the main issues is a lack of trained healthcare providers. This is especially true in India where despite efforts to try to improve healthcare, there are not enough healthcare professionals to meet the needs of the rural areas and disproportionately even fewer anesthesiologists. This deficiency can make providing safe surgical care challenging [2–5].

In India, there are reportedly only 1.27 anesthesiologists/100,000 population. This is far below the recommended 5/100,000 population recommended by the World Federations of Societies of Anesthesiologists (WFSA). This ratio is further skewed as the majority of anesthesiologists in India are reluctant to work outside urban areas [5, 6]. Unlike many other areas of the world where physician and non-physician healthcare workers are trained to provide safe anesthesia and often work in the rural settings, in India only anesthesiologists are allowed to provide anesthesia care. Efforts are being made to increase the number and distribution of anesthesiologists in India; however, short-term solutions are needed to ensure safe anesthesia is available for emergency surgical care especially in the rural areas [7–9].

For many orthopedic procedures, regional anesthesia (RA) is a viable alternative to general anesthesia (GA) [10]. While not optimal, RA administered by non-anesthesiologist physicians (NAP) with focused training and standard proficiency is reportedly associated with a low risk of complications; however, safety and efficacy remain under-evaluated [11–13]. This study audits a single surgeon’s experience providing RA and orthopedic care for emergency lower-limb musculoskeletal disorders (LL MSDs) in a low-resource setting (LRS), in the absence of an anesthesiologist.

## Material and methods

A retrospective audit of surgical and anesthesia complications performed under RA for emergency surgery for LL MSDs was conducted after ethical approval from institutional board review (IRB Min No. 13279) by Christian Medical College, Vellore (8/26/2020). All procedures were performed at Christian Hospital, Mungeli, a small mission hospital located in rural central India from July 2011 to June 2016, which is attached to Christian Medical College, Vellore. Procedures on patients less than 14 years, elective procedures, and those with anesthesia provided by an anesthesiologist were excluded.

During this period, this was the only hospital with a qualified practicing orthopedic surgeon for over 3000 square kilometers (catchment area > 700,000 people). Ninety percent of the population reside in rural areas and over 70% of households belong to the lowest or second wealth quintiles [14–16]. Only one-tenth of villages are located within 10 km of a government-run first-level referral unit (FRU) and the nearest FRU for orthopedic trauma management is the tertiary referral center nearly 100 km away. Road connectivity in this rural district is predominantly via unsurfaced roads and dependent on erratic daytime public transport and limited ambulance service [17, 18].

A protocol for administration of RA by the surgeon for emergency LL MSDs when an anesthesiologist was not available, was developed by the surgeon (Table 1) in collaboration with 3 anesthesia colleagues. One had previously worked at the mission hospital while the other two had outreach experience. They all understood that there were times in which emergency surgeries were required and a local anesthesiologist would not be available. The surgeon had received 6 weeks of basic training in anesthesia including advanced airway techniques, administration of spinal anesthesia, and basic nerve blocks during his orthopedic residency. The remainder of his RA training was informal and included a mix of instructions (via phone, face-to-face) from anesthesia colleagues, videos, YouTube, and self-study (during his mission hospital tenure). There was no real-time consultation from local anesthesiologists available. As part of the protocol, patients who required emergency essential surgery for LL MSDs were given the option to have their surgery done at the local facility with a surgeon administered RA or referral to the regional center. Per the protocol, in high-risk cases a pre-operative consultation was obtained by the surgeon from an off-site anesthesiologist whether to offer RA or recommend transfer. High-risk cases were identified through a pre-anesthetic checklist (PAC). Patient monitoring was performed as per protocol (Table 1).

**Table 1:** Protocol for NAP administration of regional anesthesia for emergency lower-limb orthopedic procedures in absence of an anesthesiologist

Pre-operative	Intra-operative	Post-operative
*Pre-operative checklist (PAC)* History and physical Examination, clinical and radiological assessment by surgeon CBC, blood borne virus screening, T&S Additional tests for patients with comorbidities or > 40 years of age (chest radiograph/ serum creatinine/ LFT/ ECG/ PT/INR) Offer surgery locally with RA by surgeon vs referral For high-risk cases, remote pre-anesthetic consultation as needed to determine whether to offer RA vs. refer Documentation of patient decision in chart Obtain written informed consent from patient Pain management with IM Opiates, NSAIDs Additional laboratory evaluations/ T&C for whole blood as needed *Pre-operative protocol* 18 G IV access 2nd IV access or central venous access if in shock/sepsis Preload with 500 ml of 0.9% normal saline (NS) (additional 500 ml administered after block placement) Premedication IM pentazocine (0·5 mg/kg, max dose 60 mg) IV ondansetron (0.1 mg/kg, max dose 8 mg) Urethral Catheterization (as indicated) IV cloxacillin 50 mg/Kg IV gentamicin 3 mg/Kg (in 100 ml NS)	*Monitors* Blood pressure, heart rate, SpO_2_ saturation, and continuous electrocardiogram (ECG)) q 5 min for first 30 min after block placement and then q15 mins Emergency airway equipment available *Spinal Block* Patient in sitting position / lateral position with hips in maximum permissible flexion 25G (or 22G) spinal needle used under strict aseptic technique Subarachnoid space confirmed by aspiration of clear spinal fluid at 3rd lumbar interspace Bupivacaine (0.5%, 3 – 3.6 ml) Assessment of sensory level & adequacy of motor paralysis with spirit-soaked cotton swab Surgical drapes after desired sensory level achieved *Failed SA* Wait for 30 min after administration of spinal block If No effect, repeat RA protocol at same or higher lumbar interspace Lidocaine (2%, 2 ml) Monitoring by ANM/GNM nurse trained in basic life support Documentation of vital signs Administer adjuvant medications as instructed by surgeon Airway management and management of any complications as per surgeons’ instruction Adjuvant intraoperative medications Per surgeon’s orders Medications include IM/IV Opiates, IM/ IV NSAIDS, IM/IV ketamine Local infiltration of lidocaine (1%, 5 – 10 mg/kg) Decision for transfusion by surgeon *Peripheral nerve block (Cocktail)* Bupivacaine (0.25%, max dose of 2 mg/Kg with 1 ml of 1:200,000 epinephrine) Lidocaine (2%, max dose of 5 mg/kg with 1 ml of 1:200,000 epinephrine)	Transferred from OR to recovery after confirming vital parameters stable (HR, RR, BP, SpO_2_) *Recovery room* Monitored by ANM / GNM nurse Documentation of vital parameters and medications q 30 min Monitoring with pulse-oximeter, automatic BP cuff and ECG leads Airway management and management of any complications as per surgeons’ instruction Pain management with IM / IV Opiates, IM/ IV NSAIDS per surgeon’s orders Transfer to ward when patient stable Surgeon's sign and date

Conversion to GA was not feasible in the given circumstances. If SA wore off before completion of the surgical procedure or PNB was not sufficient, anesthesia was augmented with intravenous midazolam, ketamine, and/or local infiltration of lidocaine. This was directed by the surgeon but administered by the person responsible for monitoring the patient. Surgical intraoperative complications were defined as equipment malfunction or breakage, surgical device failure, lack of appropriate orthopedic implant, unanticipated surgical events, technical difficulty, or errors leading to deviation from the surgical plan.

‘Anesthesia-related complications’ (ARC) were defined in relation to administration of RA and/or adequacy of the block for the surgical procedure. Complications related to administration included: difficulty with placement of RA (2 or more attempts), hypotension (systolic blood pressure < 90 and/or diastolic blood pressure < 60), bradycardia (HR < 50 beats/minute), ‘high spinal’ with respiratory distress (requiring airway support); delay in recovery from RA, and persistent neurological deficits. The block was deemed inadequate if there was incomplete sensory or motor relaxation, tourniquet pain, or failed SA requiring additional anesthetic medications for amnesia/analgesia.

A risk assessment was performed based on the numbers of ARCs that resulted in either adjuvant medications being administered and/or a change of surgical plan due to inadequate RA administered by the surgeon.

## Results

Over the five-year audit period, 766 patients with LL MSDs underwent emergency essential surgery. Patient demographics and type of surgeries performed are reported in Table 2. An anesthesiologist was only available for 6 of these procedures which are not included in this audit. Two hundred and eighty-three procedures required RA which was performed by the surgeon. SA was administered in 267 (94.3%) of these patients and sciatic, femoral, and ankle PNBs were administered in one, two, and 13 patients, respectively. The remaining 477 procedures were performed with ketamine and/or midazolam sedation administered by a nurse or medical doctor, and/or local infiltration by the surgeon. Intraoperative surgical complications were encountered in 17 patients (Table 3). ARCs were encountered in 46 patients who were administered SA and in one patient who underwent sciatic PNB. Difficulty with placement was encountered in 37 patients with an average of 2 attempts. SA failed in 9 patients and was successfully re-administered with 2% lidocaine. Transient post-spinal hypotension was encountered in 104 patients. In all cases, hypotension resolved with 0.9% normal saline boluses and rarely Mephentermine IV was needed. This was managed by the person monitoring the patient with direction by the surgeon. Since the transient hypotension quickly resolved with fluids and did not impact the surgical plan, it was not included as an ARC. No cardio-respiratory complications were noted (Table 3).

**Table 2:** Demographics of patients requiring regional anesthesia for emergency surgery for “essential” lower-limb musculoskeletal disorders (*N* = 283)

Median age (years; range)	35 (14–80)
Gender	
Male Female	213 (75.2%) 70 (24.8%)
Median duration of surgery (minutes; IQR)	120 (120–180)
Diagnosis	
Trauma (*N* = 210)	
Open injury	83 (39.5%)
Closed injury	122 (58.1%)
Compartment syndrome	3 (1.4%)
Morel Lavallee lesion	2 (0.1%)
Musculoskeletal infection (*N* = 63)	
Septic arthritis	15 (23.8%)
Acute osteomyelitis	29 (46%)
Post-operative infections	9 (14.3%)
Pyomyositis	6 (9.5%)
Gas gangrene	2 (3.1%)
Others	2 (3.2%)
Tumors (*N* = 10)	
Malignant	3 (30%)
Benign	7 (70%)
Types of essential surgical procedures performed (DCP3)^*^	
Fracture reduction	103
Management of non-displaced fracture	278
Irrigation and debridement of open fracture	83
Placement of external fixator	64
Fasciotomy	3
Trauma related amputations	3
Skin grafting	26
Drainage of septic arthritis	15
Debridement of osteomyelitis	29
Wound debridement	389
Other procedures (not included under DCP3)	
Tumors	10
Others	18
Type of regional anesthesia	
Spinal anesthesia	267 (94.3%)
Sciatic PNB	1 (0.3%)
Femoral PNB	2 (0.7%)
Ankle PNB	13 (4.6%)

* Some cases required combination of multiple DCP3 procedures

**Table 3:** Intraoperative complications

Regional anesthesia complications	
Spinal anesthesia (*N* = 267)	
Difficulty in administering	37 (13.8%)
Failed anesthesia	9 (3.7%)
Re-administration	9 (3.7%)
Need for augmentation (ketamine/sedation)	5 (1.8%)
Hypotension	104 (40%)
Sciatic PNB (*N* = 1)	
Failed anesthesia	1 (100%)
Need for augmentation (ketamine/sedation)	1 (100%)
Femoral PNB (*N* = 2)	
No complications	
Ankle PNB (*N* = 13)	
Need for augmentation (ketamine/sedation)	1 (7.7%)
Intraoperative surgical complications	17
Device failure	
Image intensifier malfunction	2
Instrument/jig breakage	4
Fracture table attachment breakage	1
Implant not available	3
Technical difficulty and mal-reduction	5
Implant pull-out requiring Re-operation	2

One patient with a closed femoral shaft fracture with severe mitral stenosis where SA was contraindicated was offered surgery under sciatic and femoral PNBs. However, anesthesia was inadequate for the patient to undergo intramedullary nailing. Hence, the surgical plan was revised to closed reduction with balanced traction [19].

SA required augmentation in 5 patients. In one of these patients, with ipsilateral femoral shaft and neck fractures, the surgical time exceeded 300 min.[20] No anesthetic complications were encountered with femoral and ankle PNBs (Table 3).

## Discussion

The prevalent practice of mandatory service obligation period after completion of postgraduate orthopedic training in India aims to address the skewed rural–urban distribution of orthopedic healthcare resources. For nearly a century, Christian Medical College Vellore has addressed this inequitable healthcare distribution by sending out providers to sustain and support healthcare in remote areas across the country. This commitment has ensured at least a minimal level of orthopedic surgical care in several remote areas [2, 3]. Evolving socio-economic status and increased healthcare demands from the rural population, push for institutional accreditation, increased standards for obtaining Government health insurance coverage and more stringent supervision by regulatory bodies have led to an emphasis on quality-oriented, specialty driven approach without fully considering the realities and challenges of delivering safe surgical care in rural areas [1, 4–6]. This orthopedic clinic comprising of a physiotherapy and occupational therapy unit, a radiology suite with an X-ray machine, an Image Intensifier, the sole CT scan machine in the entire district, and operation theater equipped with only basic orthopedic equipment and sterilization facilities served more than 7 million people (> 90% from rural areas).

Delivering safe and quality bellwether surgical care, in rural India is challenging. Recently trained orthopedic surgeons with limited experience, functioning as a single-doctor unit to provide care without an anesthesiologist is a routine narrative not just in India, but in many LMIC healthcare systems [6, 18, 21, 22]. Considering the projected shortfall and estimated time necessary to achieve the minimum goal of ensuring 20 Surgeons, Anesthesiologists and Obstetricians (SAOs)/100,000 population, rural areas will continue to have a shortage of physician anesthesiologists and will have to choose between declining care or providing emergency essential surgical care without an anesthesiologist or training a physician to give safe anesthesia without reaching full specialist status as they do in Australia and Canada [21, 23, 24]. The debate over non-physician anesthesia providers (NPA) empowerment remains controversial with skewed focus on difficult scenarios and poorer outcome but ignoring the fact that, not being able to provide emergency essential surgeries immediately to patients can result in worse outcomes for patients [13, 25–31].

Regional anesthesia is traditionally believed to be safer than GA. However, RA is not devoid of complications. To be performed safely it requires training, protocols must be followed and should be used in appropriate situations [11, 31–33]. Pawa et al. outlined the role of NAPs, and surgeons administered RA through additional training and guidelines, thus optimizing available resources to make safe anesthesia more accessible. Conversion to GA, advanced airway management, and managing unexpected complications are potential pitfalls [11]. Lewis et al. found inconclusive evidence to prove anesthesia delivered by NPAs was in any way inferior or more dangerous than when delivered by an anesthesiologist in over 1,500,000 patients in the USA [30]. Unlike the USA, most LMICs do not have specific guidelines or dedicated training for NPA; therefore, these results must be interpreted judiciously. Several studies have documented the relative safety of RA when administered by a NAP for emergency obstetric surgery where there is no anesthesiologist [29, 34, 35]. Lokossou et al., in a review of anesthesia services across 17 Sub-Saharan countries, have emphasized the training of NPAs to compensate for the critical lack of anesthesiologists [36]. Enright has encouraged increasing output of trainees from anesthesiology residency programs for rural posting as well as supervision and continued medical education for NPAs to overcome the deficit [37]. In contrast, Khan et al. have proposed a more equitable distribution of existing anesthesia workforce to ensure at least bellwether surgical procedures are carried out safely [38].

The present audit proposes NAP networking with off-site anesthesiologists for remote pre-anesthesia consultation in high-risk cases, development of NAP administered RA protocol and additional training is feasible. The role of a detailed pre-operative assessment, judicious patient selection, remote consultation and guidance from anesthesiologist colleagues, constructive team dynamics and reasonable skills to plan and execute surgical procedure promptly cannot be over-emphasized.

Surgical complications noted in this audit could be attributed either to the relative inexperience of the surgical team or limited resources. ARCs audited in this study are unique as they are determined from the surgeon’s perspective in terms of ease of administration, adequacy of muscle relaxation, appropriateness of level, duration of action, and post-operative recovery. SA was administered in a sitting position as most patients could not be turned to the side due to pain. Hence, the need for multiple attempts for SA may have resulted from suboptimal positioning rather than inexperience. A SA failure of 3.7% observed in this study is comparable to the reported 4% failure rates among anesthesia trainees but higher than the reported < 1% failure rates among experts [39, 40]. A detailed analysis of the cause of failure of SA is beyond the scope of this study. Hypotension is a common side effect of spinal anesthesia occurring in 16–33% of cases [41]. While it was detected in 40% of our patients, it was expected and easily treated with fluids and from the surgeon’s perspective did not result in any deviation from the perioperative protocol or surgical plan. For this reason, it was not included as an ARC. Lack of a real-time anesthesiologists’ perspective of ARCs is an obvious limitation in this audit but then it would contradict the very need of this audit to assess the safety and efficacy of surgeon administered RA.

Though no conclusions can be drawn regarding the few PNBs audited in this study, it does show why clinical exposure and training of non-anesthesiologists in common PNB techniques should be explored [24, 34, 37]. The unique ‘hands-on’ training during an elective clinical posting in anesthesiology may have contributed to the successful administration of SA in this case and is a testament to our institution’s commitment to training physicians for rural mission service.

This study highlights the concept of networking and remote pre-anesthesia consultation with off-site anesthesiologists to mitigate risk and provide safe anesthesia services in LRS. The rapid expansion of mobile network and familiarity with android based communication apps has made remote consultation with an off-site anesthesiologist feasible and cost-effective. However, this audit is inadequate to ascertain efficacy of remote pre-anesthesia consultations.

## Limitations

This retrospective audit suffers from inherent selection bias, lack of standardized documentation, and a universal outcome grading system. Safety and efficacy of surgeon administered RA in this cohort cannot be compared with anesthesiologist administered RA and lack of an alternate benchmark for comparison poses a significant limitation in extrapolating our results to other low-resource settings. The results of this study cannot be generalized and must be interpreted based on local resources and limitations. In addition, the technical expertise of SA and PNB are highly variable and operator dependent. Effect of spinal needle gauge, patient positioning, demographic factors, and effect of the learning curve of the NAP on results were not assessed. Finally, this study is unable to quantify stress associated with delivering essential orthopedic surgical care without an attending anesthesiologist and its impact on surgical outcomes.

## Conclusion

Regional anesthesia, especially SA proved to be an acceptable alternative for providing emergency and essential surgical care for LL MSDs in this low-resource setting, when there was no anesthesiologist. This solution must be explored further to solve the current crisis of anesthesiologist-deficit in LMICs. A qualified attending anesthesiologist cannot be replaced in a surgical team, and this study by no means implies or suggests that. Alternative measures including networking, ‘remote pre-anesthetic consultation’ and training of NAP in basic RA techniques in delivery of safe surgery need further evaluation.
